# Separation Technique for the Determination of Highly Polar Metabolites in Biological Samples

**DOI:** 10.3390/metabo2030496

**Published:** 2012-08-16

**Authors:** Yusuke Iwasaki, Takahiro Sawada, Kentaro Hatayama, Akihito Ohyagi, Yuri Tsukuda, Kyohei Namekawa, Rie Ito, Koichi Saito, Hiroyuki Nakazawa

**Affiliations:** Department of Analytical Chemistry, Faculty of Pharmaceutical Sciences, Hoshi University, 2-4-41 Ebara, Shinagawa-ku, Tokyo 142-8501, Japan

**Keywords:** capillary electrophoresis, gas chromatography, high performance liquid chromatography, highly polar metabolite

## Abstract

Metabolomics is a new approach that is based on the systematic study of the full complement of metabolites in a biological sample. Metabolomics has the potential to fundamentally change clinical chemistry and, by extension, the fields of nutrition, toxicology, and medicine. However, it can be difficult to separate highly polar compounds. Mass spectrometry (MS), in combination with capillary electrophoresis (CE), gas chromatography (GC), or high performance liquid chromatography (HPLC) is the key analytical technique on which emerging "omics" technologies, namely, proteomics, metabolomics, and lipidomics, are based. In this review, we introduce various methods for the separation of highly polar metabolites.

## 1. Introduction

Recent advances in genome sequencing have underscored the fact that our knowledge of gene function is still limited, with typically 30%–40% of open reading frames having no known function to this day [1]. In life sciences, there is an obvious need to determine the biological function of the so-called orphan genes, some of which may be molecular targets for therapeutic intervention. The search for specific mRNAs, proteins, or metabolites that can serve as diagnostic markers has intensified, as has the fact that these biomarkers may be useful in monitoring and predicting disease progression or response to therapy [2,3,4]. Metabolomics has the potential to fundamentally change clinical chemistry and, by extension, the fields of nutrition, toxicology, and medicine. Functional analyses have become increasingly popular. They include investigations at the level of gene expression (transcriptomics), protein translation (proteomics), and more recently, the metabolite network (metabolomics).

As with transcriptomics and proteomics, the analytical tools employed in metabolomics can yield massive data sets. The main obstacle in metabolomics studies is that the discovery phase is most commonly undertaken by mass spectrometry (MS) [5,6,7,8,9] or nuclear magnetic resonance (NMR) spectrometry [10,11]. MS and NMR are among the most important emerging technologies in metabolomics, offering the shortest route toward metabolite identification and quantification [12]. NMR has demonstrated great potential, essentially due to the high measurement reproducibility and the high throughput of analysis [13,14]. However, one major problem in metabolomics studies by NMR is the relatively poor sensitivity of the technique. Furthermore, the number of MS researchers is much larger than that of NMR researchers trained to acquire metabolomics data [15]. In parallel, capillary electrophoresis (CE) [16], gas chromatography (GC) [17], and high performance liquid chromatography (HPLC) [18,19,20] separation techniques coupled to online MS detection can provide higher levels of sensitivity.

Many important endogenous metabolites exist at very low concentrations in biological systems. Metabolomics could enable mapping of perturbations of early biochemical changes in diseases and hence provide an opportunity to develop predictive biomarkers that could result in earlier intervention and provide valuable insights into the mechanisms of diseases. The primary goal of metabolomics analysis is the unbiased relative quantification of every metabolite in a biological system. Organic acids and amino acids represent metabolite classes of high significance because these metabolites are involved in a multitude of biochemical pathways and fluxes and are thus important for the diagnosis/evaluation of a number of critical metabolic states. However, these metabolite classes can be difficult to separate from each other and matrix components because of their polar nature.

In this review, we introduce various separation methods, such as CE, GC, and HPLC, for the determination of endogenous highly polar metabolites in biological samples.

## 2. Non-Target and Target Metabolomics

Metabolomics is a promising approach aimed at facilitating our understanding of the dynamics of the biological composition in living systems. Metabolomics is classified into non-targeted or targeted metabolomics. Non-targeted metabolomics seeks to assign as many compounds as possible by either *de novo* analyte identification or ideally, utilizing reference standards to achieve the highest level of confidence. Changes in the metabolites can be mapped to specific pathways, thereby providing mechanistic information of the process under study. Targeted metabolomics measures analytes that have been selected *a priori*, on the basis of known biology and chemistry. Thus, targeted metabolomics is similar to conventional multiplexed bioanalytical assays. Metabolomics is employed in studies of cancer [21,22,23], diabetes [24,25], plants [26,27], drugs [28,29], and biomarkers of several diseases [30,31,32].

Sample pretreatment for metabolomic analysis depends on non-targeted or targeted study. For non-targeted metabolomics, it is desirable that the biological sample is analyzed with minimal pretreatment to prevent the loss of metabolites. For targeted metabolomics, deproteinization of the biological sample is often followed by off-line solid phase extraction (SPE), which is used for sample desalting and preconcentration of the target metabolites from the sample matrix. However, highly polar metabolites do not show retention on commonly used SPE columns and elute simultaneously with the salts.

A major obstacle in metabolomics remains the identification and quantification of a large fraction of unknown metabolites in complex biological samples when purified standards are unavailable. Generally, metabolite identification or confirmation is based on accurate mass, retention time, and fragmentation patterns, using standards and databases [33]. Hence, most metabolomics researchers experimentally compare the MS/MS pattern of a model compound to that of the putatively identified molecule from the research sample. Metabolite quantification and identification is still a highly challenging task in non-targeted metabolomics studies.

## 3. Separation Technique of Highly Polar Metabolites

### 3.1. Capillary Electrophoresis

Different methodologies offer distinct advantages that can be exploited in order to investigate in detail a variety of metabolite classes, and the resulting information is accumulated to better characterize a particular metabolome. Complementary approaches are of utmost importance. In this regard, CE-MS definitely has a place in metabolomics research [34]. 

CE is a separation technique that is based on the differential transportation of charged species in an electric field through a conductive medium. CE has a number of separation modes, such as capillary zone electrophoresis (CZE), capillary gel electrophoresis (CGE), capillary isoelectric focusing (CIEF), micellar electrokinetic chromatography (MEKC), electrokinetic chromatography (EKC), and non-aqueous capillary electrophoresis (NACE). CE is versatile in that it enables separation of a wide range of analytes, from small inorganic ions [35] to large proteins [36]. The separation conditions (capillary length, buffer ionic strength, pH, and viscosity) have a direct influence on the intensity of electroosmotic flow (EOF). MEKC is a powerful tool for separating neutral compounds based on their partition to charged micelles [37]. In MEKC, various chiral surfactants, including polymerized surfactants, were developed for the enantioseparation of amino acids [38]. However, CE cannot be combined with MS in a straightforward way because micelles tend to contaminate the ion source, suppress analyte ionization, and decrease MS response. CE buffers are generally aqueous systems, although nonaqueous systems are used as well, particularly for analytes that are insoluble or sparingly soluble in water. Moreover, they enable better exploration of certain hydrophilic interactions, such as hydrogen-bonding, dipole-related, and ionic interactions, which are thermodynamically strengthened in a hydrophobic environment [39].

Since its initial trials in the mid-1980s and its first presentation in 1987 [40], CE has been coupled online to MS. ESI is the softest ionization technique currently available [41]. It transforms ions in solution into ions in the gas phase prior to MS. Although borate- or phosphate-based electrolytes have been shown to provide highly efficient CE separation, such systems are not amenable to MS [42]. The lack of volatility of borates generates an unstable ESI signal because of tip clogging and considerable ion suppression. Significant improvements in spray stability were obtained by decreasing the borate-based buffer concentration. On the other hand, such a procedure is not applicable to highly complex mixtures, since low-electrolyte concentrations result in a severe deterioration of the CE separation efficiency. As an effect of these technical problems and contradictory conditions for separation and detection, only a limited number of methodologies for online CE-MS implementation in biological analysis have so far been developed. When hyphenated with MS via ESI, NACE circumvents buffer compatibility problems and even enhances the sample ionization process to result in improved detection limits compared to separation in aqueous buffer systems [43]. Bianco *et al*. [44] used NACE to determine glycoalkaloids in *Solanum tuberosum* (potato), and the results were comparable to those obtained using LC-MS. In most biomedical and clinical metabolomics studies, CE-MS has been mainly used as a profiling method [45]. All CE-MS-based metabolomics studies have been performed with ESI, which is suitable for the analysis of highly polar compounds [46,47]. The above separation strategies are the basis of many studies aimed at furthering our understanding of mixtures, such as proteomics [48], metabolic profiling, or metabolomics [49], particularly when those strategies are used in combination with MS and such soft ionization techniques as ESI. CE-MS applications are summarized in Table 1. 

**Table 1:** Summary of CE-MS applications in metabolomics.

Sample	Analyte	Separation method	Sample preparation	Separation condition	Reference
Rat urine	Non-targeted	CD-MERK	Filtered urine	25 mM sodium tetraborate decahydrate, 75 mM SDS and 6.25 mM sulfated β-cyclodextrin. The pH was adjusted to pH 9.50 (apparent) with 2 M NaOH (after addition of SDS and cyclodextrin) with 2.25% v/v hexafluoroisopropanol.	[50]
		CZE			
Red blood cell	Amino acid	CE-ESI-MS	Prepared in 200 mM ammonium acetate	1.4 M formic acid (pH 1.8)	[51]
Tumor tissues (colon and stomach)	Anionic, cationic metabolites and nucleotide	CE-ESI-time-of-flight mass spectrometry (TOFMS)	Homogenized in water. Liquid-liquid extraction with water and chloroform. Filtered through a 5-kDa cutoff filter.	Anionic metabolites: 50 mM ammonium acetate	[52]
				Cationic metabolites: 1 M formic acid	
Neurons ( *Aplysia californica*)	Neurotransmitters	CE-ESI-TOFMS	After the protease treatment, ganglia were washed in artificial seawater.	1% formic acid	[53]
Plant extract	Anionic metabolite	CE-ESI-TOFMS	The frozen leaf disc was homogenized in methanol. Liquid-liquid extraction with water and chloroform.	50 mM ammonium acetate adjusted to pH 9.0 by the addition of ammonium hydroxide.	[54]
Mouse blood and tissue	Anionic and cationic metabolites	CE-ESI-TOFMS	The frozen tissue was homogenized in methanol. Liquid-liquid extraction with water and chloroform. Filtered through a 5-kDa cutoff filter. The filtrate was lyophilized and dissolved in water.	Anionic metabolites: 50 mM ammonium acetate solution (pH 8.5)	[55]
				Cationic metabolites: 1 M formic acid	
Rat plasma	Anionic and cationic metabolites	CE-ESI-TOFMS	The plasma was plunged into methanol. Liquid-liquid extraction with water and chloroform. Filtered through a 5-kDa cutoff filter. The filtrate was lyophilized and dissolved in water.	Anionic metabolites: 50 mM ammonium acetate solution (pH 8.5)	[56]
				Cationic metabolites: 1 M formic acid	
Human HT29 colon cancer cell	Non-targeted	CE-ESI-TOFMS	SPE	1 M formic acid	[57]
			Protein precipitation		
			Ultracentrifugation		
Human urine	Non-targeted	CE-ESI-MS	Urine sample was diluted and centrifuged.	10% acetic acid (pH 2.2)	[47]

### 3.2. Gas Chromatography

GC is most useful for the analysis of trace amounts of organically extractable, non-polar, volatile compounds and highly volatile compounds. Moreover, the use of GC-MS in the scan mode allows for non-targeted metabolic profiling and the discovery of novel compounds and metabolites [58]. However, GC-MS has limitations in the analysis of highly polar compounds due to their thermolability and low volatility. For that reason, samples must be derivatized prior to separation by GC, which increases the volatility and stability of the analytes. Hydroxyl, amine, and thiol groups are derivatized by a silylating reagent to increase volatility. There are two main classes of silylating reagents: those producing trimethylsilyl (TMS) derivatives and those producing *tert*-butyldimethylsilyl (TBDMS) derivatives. TMS derivatives can be produced by a wide variety of reagents, including *N*,*O*-bis(trimethylsilyl)trifluoroacetamide (BSTFA) and *N*-methyl-*N*-(trimethylsilyl)trifluoroacetamide (MSTFA). TBDMS derivatives are prepared by the reaction with *N*-methyl-*N*-(*tert*-butyldimethylsilyl)trifluoroacetamide (MTBSTFA) [59,60]. Higher reproducibility is realized with GC-MS than with LC-MS, and better compound separation is achieved. However, the main advantages of LC-MS/MS over the classical GC-MS procedures are the reduced time of analysis due to the elimination of derivatization steps prior to the chromatographic separation, and the fact that LC-MS/MS allows us to determine the ratio of the conjugated fraction (glucuronide and sulfate) to the free fraction, thus reducing the risk of false-positive or misleading results [61].

### 3.3. High Performance Liquid Chromatography

Compared to GC, HPLC is a powerful tool for determining highly polar compounds in several matrices [62]. In particular, reversed-phase liquid chromatography (RPLC) run on an octadecyl silica column is an indispensable technique, *i.e.*, RPLC enables use of both aqueous and organic solvents.

HPLC is the most widely used analytical technique in the pharmaceutical industry because of its versatility and ability to retain and resolve a number of compounds. However, the retention of polar analytes often requires a highly aqueous mobile phase, which can cause a number of problems, such as decreased sensitivity in electrospray ionization mass spectrometry (ESI-MS) [63].

Although it is a powerful separation mode, one major limitation of RPLC is its inability to adequately retain extreme polar compounds (*i.e.*, more highly polar compounds). In particular, the major focus of untargeted LC-MS-based metabolomics studies in recent years has been to improve the separation of water-soluble compounds. In this case, normal-phase liquid chromatography (NPLC), which includes silica or alumina as the stationary phase, is used. NPLC is generally used to separate highly polar compounds with no water in the mobile phase, e.g., hexane, chloroform, or benzene, under isocratic elution conditions [64]. However, because it is difficult to dissolve hydrophilic compounds, such as peptides and nucleosides, in a non-aqueous mobile phase, the application of NPLC to biological samples is limited. On the other hand, despite the versatility of RPLC, serious retention and/or selectivity problems may arise for highly polar compounds. Ion pair chromatography offers a robust method for separating highly polar compounds, such as sugar phosphates [65], nucleotides [66], and carboxylic acids, by using an RP column.

For example, the retention of polar acids in RPLC usually requires strongly acidic eluents with a large volume of water or the addition of cationic ion-pairing additives [67]. These elution conditions may, however, affect MS-based detection by reducing the ionization yield of the target analyte [63,68].

In general, highly polar compounds can be separated by (i) hydrophilic interaction chromatography (HILIC), (ii) the addition of ion-pairing agents to the mobile phase, and (iii) the incorporation of ion ligands within conventional RP surfaces to enable mixed mode separation [33]. In particular, HILIC, which was developed by Alpert for the separation of highly polar compounds in 1990 [69], is a powerful separation technique. HILIC-MS was employed to separate and quantify highly polar compounds in biological samples [70,71,72]. In HILIC, retention increases with increasing polarity of the stationary phase and solutes and with decreasing polarity of the organic solvent systems used for elution; this contrasts with the trend observed with RPLC. Recently, HILIC was coupled to MS and used in metabolomics studies [73]. Analytical methods for biological samples were developed [74,75,76,77], the focus of which was on metabolic pathways. Metabolic profiling offers important information for interpreting the efficacy outcome, explaining the toxicity of lead compounds, and rationalizing the toxicity of specific drugs during drug discovery or development [78]. HILIC clearly provides different selectivity from RPLC for the separation of highly polar compounds [73,79]. Therefore, HILIC is useful for determining polar metabolic compounds. LC-MS applications are summarized in Table 2.

**Table 2:** Summary of LC-MS applications in metabolomics.

Sample	Analyte	Separation method	Sample preparation	Separation condition	Reference
Human plasma	Non-targeted	LC-MS/MS	Human plasma was mixed with water, extraction buffer, and extraction solvent. The sample was shaken and centrifuged; then, supernatant was transferred onto a plate, dried down under nitrogen flow, and reconstituted in acetonitrile/water (20/80) with 0.1% formic acid.	0.1% formic acid in water and 0.1% formic acid in acetonitrile	[[80]
				Acquity UPLC BEH Shield RP18 column (1.7 μm, 50 mm × 2.1 mm)	
Human urine	Non-targeted	HILIC-MS	Urine was mixed with acetonitrile before being centrifuged.	13 mM ammonium acetate buffer (pH 9.1) and acetonitrile	[81]
				Aphera NH_2_ polymer (5 μm, 150 mm × 2 mm)	
Human serum	Non targeted	LC-TOFMS	Serum was mixed with acetonitrile and centrifuged. The supernatant was lyophilized and reconstituted.	0.1% formic acid in water and acetonitrile (98:2), and acetonitrile.	[82]
				BEH C18 column (1.7 μm, 50 mm × 2.1 mm)	
*Trypanosoma brucei brucei*	Non-targeted	HILIC-Orbitrap LC-MS	Metabolites were extracted from the cell pellet by addition of chloroform/methanol/ water (1:3:1).	0.1% formic acid in water and 0.08% formic acid in acetonitrile	[83]
(strain 427)				ZIC-HILIC (5 μm, 150 mm × 4.6 mm)	
Human aortic endothelial cells	Amine	LC-MS/MS	Cell sample was lysed with a sonic dismembranator.	10 mM formic acid in water and 10 mM formic acid in methanol	[84]
				Capillary column with in-house made (3 μm, 200 mm × 0.075 mm)	
Human urine	Carnitine and acylcarnitine	LC-MS/MS	Urine sample was extracted using methanol.	Eluent A, acetonitrile/water (80/20); eluent B, acetonitrile/water (20/80); eluent C, acetonitrile/ water/acetic acid/triethylamine (20/80/0.5/0.5); and eluent D, acetonitrile/water/acetic acid/ triethylamine (90/10/0.5/0.5)	[85]
				Hypersil MOS-1 C_8_ (3 μm, 100 mm × 4.6 mm)	
Extracellular metabolites from acute lymphoblastic leukemia cell	Purine and pyrimidine metabolite	HILIC-TOFMS	T Culture medium sample was processed by adding of acetonitrile, vortexing, and centrifuging.	0.1% formic acid in water and 0.1% formic acid in acetonitrile	[86]
				Acquity amide column (1.7 μm, 150 mm × 2.1 mm).	
Human serum	Non-targeted	LC-TOFMS	Deproteinization	0.1% formic acid in water and 0.1% formic acid in acetonitrile	[87]
			Liquid-liquid extraction	Mediterranea reversed-phase C18 analytical column (3 μm, 100 mm × 0.46 mm)	
			Solid-phase extraction		
Human platelet	Non-targeted	HILIC-MS	Cell sample was extracted using several solvents.	0.1% formic acid in water and 0.1% formic acid in acetonitrile	[88]
				Acquity amide column (1.7 μm, 150 mm × 2.1 mm)	
*Saccharomyces cerevisiae*	Phospholipid	HILIC-MS/MS	Liquid-liquid extraction with methanol and chloroform. Aliquot of the upper phase was collected.	Acetonitrile/water, 70:30 (v/v) with 10 mM ammonium acetate adjusted to pH 4.5	[89]
				Xbridge HILIC (5 μm, 150 mm × 4.6 mm)	

## 4. Applications

### 4.1. Amino Acids

Amino acids are precursors for the synthesis of proteins, other nitrogenous substances, glucose, and fatty acids [90,91]. Amino acids play a major role in energy metabolism, neurotransmission, and lipid transport, and are important in disease diagnostics, and in elucidating nutritional influences on physiology [92,93]. Amino acids not only act as building blocks of proteins but also serve as key regulators of metabolic pathways in cells. However, the mechanisms responsible for the effects of amino acids are largely unknown. Metabolomics studies are very difficult to perform because of the wide variability of biological fluids (mainly urine) in association with different confounding factors, such as gender, age, time of day, state of health, lifestyle, diet, and phenotype [94]. Therefore, the integration of various omics technologies and bioinformatics with conventional techniques is expected to provide comprehensive information about amino acid metabolism and nutrition in organisms. 

Waldhier *et al*. applied GC×GC-TOFMS to the separation of amino acid enantiomers after derivatization with methyl chloroformate [95], and were able to successfully separate 10 amino acid enantiomers from serum and urine matrix. On the other hand, Williams *et al*. developed CE-MS for the metabolic profiling of amino acids [96] and concluded that CE-MS is a valid alternative to GC-MS for the targeted profiling of metabolites, such as amino acids, because it possesses significant advantages over GC-MS. Liu *et al*. applied a sensitive and precise metabolomics method based on HILIC-TOFMS to investigate metabolic changes [97]. They were able to detect biochemical changes in the mesencephalon of transgenic mouse, specifically S100B, a Ca^2+^ binding protein. HILIC-TOFMS may help improve our understanding of the mechanism of action of S100B protein in the development of Parkinson’s disease.

### 4.2. Glutathione and Related Compounds

Reduced glutathione (GSH) is a major intracellular nonprotein thiol that plays a vital role in protecting cells and tissues from oxidative injury. It is a tripeptide composed of cysteine (Cys), glutamic acid, and glycine. GSH is present in all organs, particularly the liver. It is present in virtually all mammalian tissues. GSH plays an essential role in maintaining an intracellular redox environment that is critical for the function of cellular proteins. Under oxidative stress conditions, GSH is oxidized to glutathione disulfide (GSSG) and/or bound to a protein. Therefore, the GSH to GSSG (oxidized GSH and protein-bound GSH) ratio is altered. RPLC can simultaneously detect GSH, other low-molecular-mass thiols and disulfides, but requires sample derivatization because non-derivatized GSH and GSSG are highly polar compounds and thus difficult to determine.

**Figure 1:**
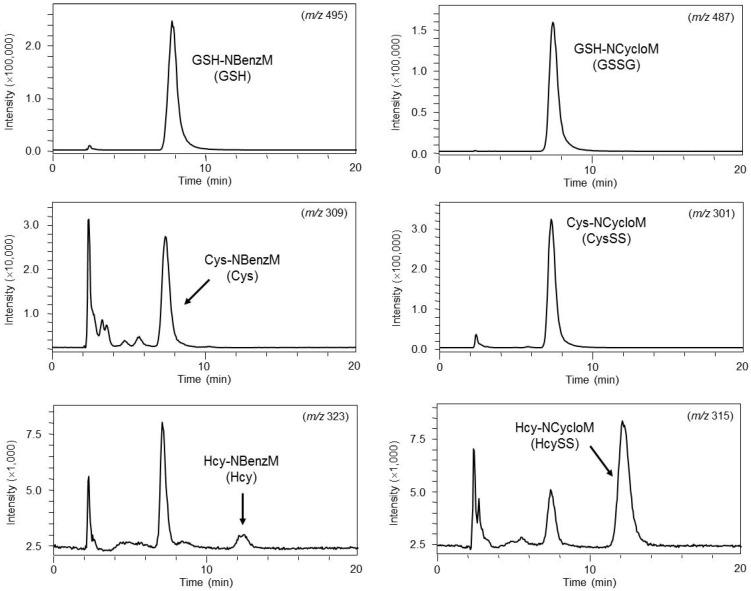
Chromatograms of reduced and oxidized thiols in mouse serum sample subjected to column-switching HILIC-MS. Adapted from reference [75].

Dernovics *et al*. confirmed the potential of electrospray quadrupole time-of-flight (QTOF) MS/MS for the identification of selenium metabolites, such as GSH conjugate, which is in the low intra scan dynamic range [98]. Iwasaki *et al*. developed a highly sensitive and accurate method that employs column-switching HILIC-MS for the determination of reduced and oxidized thiols [75]. Representative chromatograms of serum samples are shown in Figure 1. They succeed in measuring thiol compounds in mouse serum.

On the other hand, D'Agostino *et al*. developed an analytical method for thiols, which utilizes CE-MS [99]. Husain *et al*. applied CE-TOFMS to quantitate cysteine deprivation in *Entamoeba histolytica* [100]. The optimization of maleimide labeling in conjunction with online sample preconcentration allows for the simultaneous analysis of nanomolar levels of reduced thiols and free oxidized thiols. This method integrates both specific and nonspecific approaches toward sensitivity enhancement for the artifact-free quantification of labile plasma thiols without complicated sample handling. Plasma thiol redox status determination, together with untargeted metabolite profiling, offers a systemic approach for the elucidation of the causal role of dysregulated thiol metabolism in the etiology of human diseases.

### 4.3. Neurochemicals

Given the physiological importance of neurotransmitters as signaling molecules in the central nervous system (CNS), the ability to measure changes in neurotransmitter concentrations has become an integral part of CNS drug discovery and development. These neuroactive molecules and metabolites, consisting of neurotransmitters, such as glutamate, or neuromodulators, such as acetylcholine (ACh), can be detected by appropriate techniques in the extracellular fluid of the brain. Many of these neurotransmitters also exist in blood, but their concentrations are different from those in the brain because of the differential permeability of the blood-brain barrier (BBB) and of differences in absorption mechanism, synthesis, and metabolism. In recent years, the accurate measurement of neurotransmitter concentrations in an accessible matrix has provided an opportunity to use those concentrations as preclinical and clinical biomarkers of CNS penetration and target engagement [101,102]. ACh, one of the neurotransmitters released by cholinergic neurons in the CNS, plays an important role in sleep regulation, learning and memory, cognitive function, and the pathology of neurological disorders, such as Parkinson’s disease, Alzheimer’s disease, and schizophrenia. Therefore, an analytical technique that enables the simultaneous determination of biomarkers of both cholinergic and histaminergic systems in an accessible biological matrix, such as CSF, would be a useful research tool to better understand the underlying mechanisms and implications for therapeutic interventions.

Diao *et al*. developed a simple and sensitive method for the simultaneous analysis of three catecholamines: dopamine, epinephrine, and norepinephrine, in urine, by CE, coupled with in-column fiber-optic light-emitting diode-induced fluorescence detection (ICFO-LED-IFD) [103]. CE-ICFO-LED-IFD has been successfully applied to the analysis of catecholamines in human urine samples, offering good accuracy and satisfactory recovery. Meanwhile, Zhang *et al*. developed and validated a UPLC-MS/MS method to simultaneously quantify neurochemical concentrations in rat CSF. They used a HILIC column to separate highly polar compounds [104]. Li *et al*. determined neurochemicals in brain and blood samples of non-human primates in parallel by dual microdialysis, and subsequently conducted analysis by a direct capillary HILIC-MS-based method [105].

### 4.4. Tricarboxylic Acid Cycle

Studies of the metabolites in the tricarboxylic acid (TCA) cycle are considered to be essential for metabolomics analysis. The main metabolites in the TCA cycle are di- and tricarboxylic (TCA) acids. The TCA cycle has three primary functions: (i) to provide biosynthetic intermediates, (ii) to generate reducing potential, and (iii) to directly produce a small amount of ATP. The availability of biosynthetic intermediates affects the availability of amino acids and nucleic acids. Mammalian cells depend on the metabolism of glucose and glutamine for proliferation. The coupling of separation methods, such as CE [106], GC [107], RPLC [108], and HILIC [109], to MS has proven to be effective for the detection of large numbers of metabolites in complex mixtures.

Büscher *et al*. compared the three separation platforms that are most widely used in the analysis of intracellular metabolites: CE, GC, and LC, all in combination with a TOFMS detector [110]. The more limited coverage of GC is due to a bias in the detection of large polar molecules. This is caused by the derivatization that renders nonvolatile polar compounds amendable to gas-phase separation, but cannot be completed because of steric hindrance of the numerous silyl groups that are necessary to modify all amino, carboxy and hydroxy groups in large molecules. According to their conclusions, for analyses on a single platform, LC provides the best combination of both versatility and robustness. If a second platform can be used, it is best complemented by GC.

## 5. Conclusions

Metabolomics is a promising approach aimed at facilitating our understanding of the dynamics of biological composition in living systems. Metabolites tend to be converted into highly polar compounds and are therefore difficult to separate. In this review, we discussed recent progress in the separation of biological samples. CE, GC, and HPLC are powerful tools for the separation of biological samples. Methods based on chromatographic separation coupled to MS seem optimal to meet these requirements. GC-MS needs laborious clean-up and often derivatization and it can only be applied for thermally stable compounds. CE-MS and LC-MS is a suitable alternative in many cases. These techniques will be useful to bioanalytical scientists.
